# Supplementary material for: A Monosodium Iodoacetate Osteoarthritis Lameness Model in Growing Pigs
Source: Animals (Basel). 2019 Jul 1;9(7):405. doi: 10.3390/ani9070405 (PMC6680622; doi:10.3390/ani9070405)
Supplement: Supplementary file 1 [file animals-09-00405-s001.zip › Proofed_Supplementary Materials to Manuscript ID animals-529257.docx]

Supplementary Materials to Manuscript ID animals -529257

Material A: Optimization of intra-articular injection technique on cadaver material

The intra-articular injection technique was practiced diligently on cadaver material of same size piglets using toluene blue. Optimization was carried out prior to performing the pilot study (see Material B) and the main study in growing pigs involving intra-articular deposition of Monosodium Iodoacetate in the left proximal intracarpal joint,

A total of 6 left frontlegs of piglet cadavers from the experimental pig farm (unrelated to any experimental study) presented for necropsy to the Department of Pathobiology, Faculty of Veterinary Medicine, Utrecht University were obtained. The dorsal aspect of the carpal joint was presented as shown in Figure S1, for intra-articular injection of 0.25 ml of Methylene Blue (10 mg/ml, Veterinary Pharmacy, Faculty of Veterinary Medicine, Utrecht, Netherlands), diluted 1 in 3 (v/v) with normal saline.


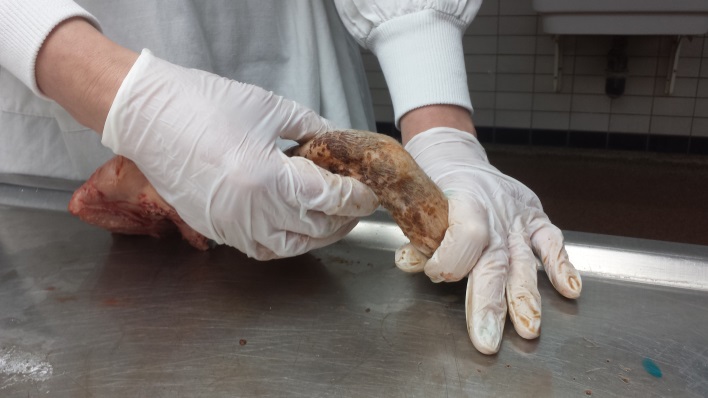


**Figure S1**. Presentation of the dorsal aspect of the left proximal carpal joint. The left carpus was slightly bended in a 100° to 110° angle, under gentle distention to maximize the dorsal intercarpal joint space.

Using firm pressure from the operator’s left hand thumb, the largest intercarpal space between the proximal and middle row of intercarpal bones was identified, just medial from the central axis. On a syringe prefilled with methylene blue solution, a 12 mm needle (26G, Sterican, Bbraun, Melsungen, Germany) was mounted and the needle was very slowly inserted resistance-free for 2-3 mm in a slightly dorsal trajectory (see Figure S2).


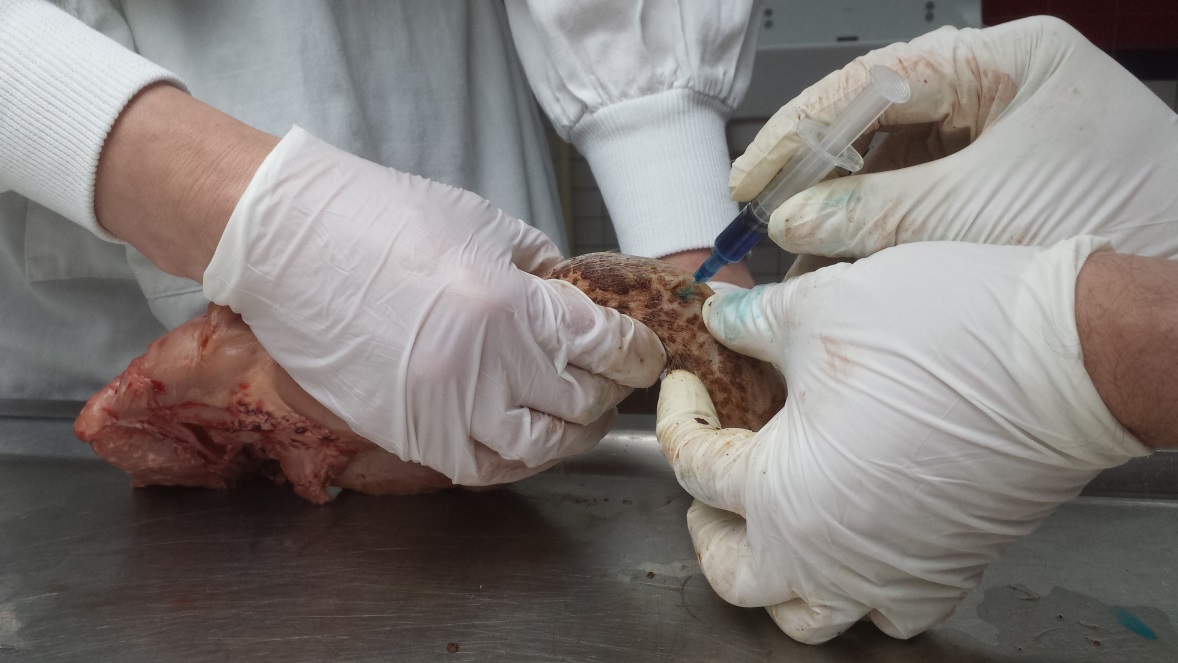


**Figure S2**. Injection site, angle and depth of needle advancement for intra-articular deposition of methylene blue into the intercarpal joint.

Once the depth of 3 mm was reached, a volume of 0.5-ml methylene blue solution was slowly injected and the needle retracted, after which the carpus was slowly flexed and distended several times to facilitate the spread of injectate throughout the joints sac.

After deposition of the injectate intra-articular deposition was confirmed by opening the carpal joint and assessing the distribution of blue dye (see Figure S3).


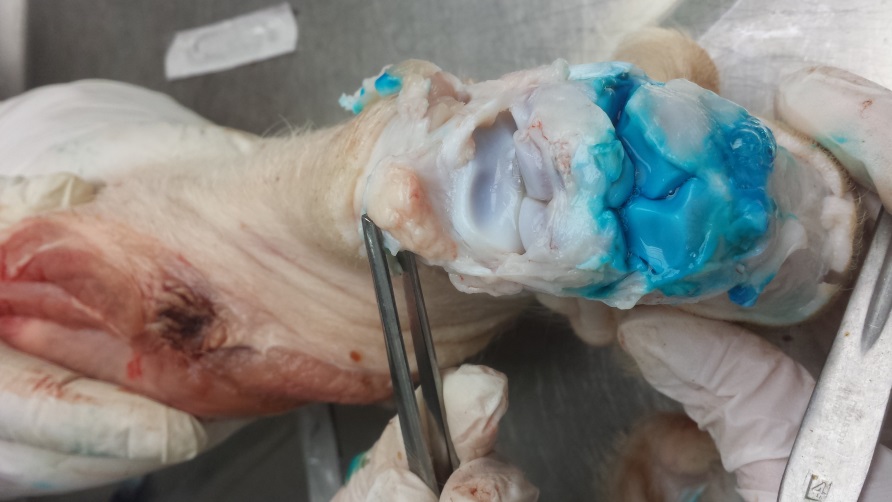


**Figure S3**. The distribution of blue dye in the carpal joint sac. Note that the dye distribution is limited to the middle intercarpal joint surfaces. The proximal intercarpal joints surfaces are not stained. The slight dorsal angle of the needle trajectory (white arrow) prevents abrasive needle trauma to the cartilage ridges indicated by the two white stars.

Material B: Pilot study: *In vivo* intra-articular Monosodium Iodoacetate dose finding study

Materials and Methods

*2.1* *Ethical statement*

The study design, including a standard analgesia protocol was reviewed and approved by the local ethical committee of Utrecht University (no. 2014.I.11.085, date of approval 17 December 2014), The Netherlands, and was conducted in accordance with the recommendations of EU directive 86/609/EEC. All effort was taken to minimize the number of animals used and their suffering. The analgesic protocol for all 8 animals consisted of 0.01 mg/kg buprenorphine IM (0.3 mg/ml, Temgesic, Reckitt Bensicker BV, Hoofddorp, The Netherlands) every 12 hours from just before intra-articular injection right up till Euthanasia.

*2.2 Animals*

At weaning at the age of 28 days, 8 piglets (Finish Landrace x Duroc cross), born at the teaching farm of Utrecht University, were selected from a total of 2 litters (Table S1). All piglets underwent a general clinical examination and visual assessment of locomotion by a veterinarian at the time of recruitment. Only healthy, sound piglets were selected. At 32-days of age the selected piglets (4 per litter) were transported to the research facility of the Department of Farm Animal Health, Faculty of Veterinary Medicine, Utrecht University.

**Table S1.** Litter of origin, gender and treatment group of selected piglets.

| **ID** | **Litter** | **Gender** | **Treatment** |
| --- | --- | --- | --- |
| 1 | 1 | M ^1^ | 20 mg MIA ^3^ |
| 2 | 1 | M | SC ^4^ |
| 3 | 1 | F ^2^ | 10 mg MIA |
| 4 | 1 | F | 40 mg MIA |
| 5 | 2 | F | 40 mg MIA |
| 6 | 2 | M | 10 mg MIA |
| 7 | 2 | F | 20 mg MIA |
| 8 | 2 | F | SC |

^1^ M=Male; ^2^ F=female; ^3^ MIA = Monosodium Iodoacetate treatment; ^4^ SC = Saline Control

*2.3 Housing*

Piglets were group-housed with littermates in 2 pens (250 cm x 250 cm; 4 animals per pen) in a single experimental room Agroflex protect rubber trowel floor (Inter Continental B.V., Ijsselsteyn, The Netherlands) with a thick layer of sawdust and an abundant straw bedding on top. In each pen additional enrichment consisted of a metal chain, plastic ball, 2 rubber chewing sticks and a think twined rope. Water was available *ad libitum* through a drinking nipple. The pigs were fed commercial standard food for weaned piglets (Prevent 3 Kruimel, de Heus, Ede, The Netherlands) *ad libitum* in a large food trough. The stable was climate controlled with a relative humidity of 55% ± 10% and a stepwise decreasing temperature regime, starting at 26 ˚C for the first 3 days and decreasing it by 0.5 ˚C every second day down to 21 ± 0.5 ˚C, at which it was maintained for the remainder for the study. In the first week one heat lamp per pen was provided for additional temperature support. The room had a 12-hours on, 12-hours off artificial light regime, with lights on starting at 7 a.m. A radio was playing softly in the room day and night, including during training and habituation.

*2.4 Handling and habituation*

All husbandry related activities and general welfare checks were performed daily by qualified personnel that the pigs were familiar with. Piglets were handled daily by the researchers and were habituated to the experimental set-up in progressively smaller groups, until animals were able be alone in the experimental set-up 3 weeks later. To minimize the time needed to collect successful trots for open space locomotion videotaping as well as the for a pressure mat runway, the pigs were trained before the start of the experiment using positive reinforcement with clickers and sugar-coated milk chocolate treats (M&M’s®, Mars Chocolate, Veghel, Netherlands) as rewards. Normal exploratory behavior across the runway was shaped until each pig was able to trot down the runway in a straight line at constant speed without stopping.

*2.5 Experimental design*

Piglets from each litter were randomly assigned at day 0 (envelope method; n=1 per treatment per litter) to either one of the MIA dose treatments (MIA-10 [n=2]; MIA-20 [n=2] or MIA 40 [n=2]; corresponding with 10, 20 and 40 mg of MIA) or the isotonic saline control group (SC-group [n=2]; All personnel, with the exception of the researcher performing the intra-articular injections (JU) remained blinded for treatment.

Pressure mat measurements as well as videos of the locomotion of the pigs were collected on several timepoints (Table S2). Visual scoring was performed off-line from videos to keep the observers blinded with respect to the timepoint and group.

2.5.1 Intra-articular injection

Intra-articular injection technique was practiced with Methylene Blue on post-mortem material of pigs of the same size as the experimental animals (see Materials A: Optimization of intra-articular injection technique on cadaver material)

Anaesthesia, aseptic intra-articular injections and recovery were all carried out in dedicated rooms separated from the housing room. At day 0, animals were guided from their housing pen into a dedicated pen for induction of anaesthesia. Animals were sedated 2 at a time with dexmedetomidine IM (15 µg/kg, Dexdomitor 0.5 mg/ml, Orion Pharma, Finland). Following clinical sedation, general anaesthesia was induced using ketamine (10 mg/kg, Narketan 10%, Vétoquinol S.A., France) and midazolam (IM; 0.5 mg/kg, Midazolam Actavis, 5 mg/ml, Actavis Group PTC, Iceland) in the same syringe. Under general anaesthesia, the animal was transported to the procedure room. An IV cannula (22 G, Terumo Surflash , IMS (Euro) Ltd, Cheadle Heath, UK) was placed in an auricular vein. An Isotonic saline drip (BBraun, Melsungen, Germany) at a rate of 5 ml/kg/hour was commenced. A dose of 10 μg/kg buprenorphine IM was administered and supplemental flow by oxygen (5 L O_2_/min) was provided during the procedure and in recovery. The piglet was wrapped in reflective foil from mid-thorax down to limit heat loss. A dose of propofol IV (0.5-1 mg/kg; Propofol 10 mg/ml, Fresenius Kabi, Huis ter Heide, Netherlands) was administered if clinical depth of anaesthesia was judged to become light. The left carpal joint was aseptically prepared by clipping, followed by a 2 -step removal of hair and debris using an iodine-based scrub solution, and disinfection with 70% alcohol and chlorhexidine (Hibisol, Regent Medical Ltd., UK). Pigs received a slow intra-articular injection of 0.5 ml, containing either MIA (80 mg/ml or diluted with sterile water for injection to 40 or 20 mg/ml respectively, sodium iodoacetate BioUltra 98%, Sigma Aldrich, USA), or sterile NaCl 0.9% (B. Braun Melsungen AG, Germany). The left carpus was slightly bended in a 100° to 110° angle, under gentle distention, and the needle was inserted dorsally, just medial from the central axis, 2 to 3 mm deep, into the proximal intercarpal joint. After deposition of the injectate, the carpus was slowly flexed and distended, before animals received atipamezole (IM; 0,3 mg/kg Atipam 5 mg/ml, Eurovet Animal Health, Netherlands) and were transported to a holding area dedicated for recovery. Soft flooring and external warmth were provided until animals were able to walk back to their housing pen.

**Table S2.** Experimental procedures.

| **Day relative to joint injection** | **Age of animals (days)** | **Performed procedures** |
| --- | --- | --- |
| -22 | 28 | Selection of litters and piglets; Weaning |
| -19 | 31 | Transport to the experimental stable and housing with littermates in a pen |
| -19 to -3 | 31-45 | Habitation and training (to personnel, being alone in the experimental runway and even trots in the runway) |
| -3 | 45 | Baseline measurements pressure mat measurements   - Video recording of gait - Pressure mat measurements |
| 0 | 48 | General anaesthesia for intra-articular injection:   - MIA (6 animals, 2 ♂^1^, 4 ♀^2^) |
|  |  | - Isotonic saline (2 animals, 1 ♂, 1 ♀) - Daily IM administration of 10 ug/kg buprenorphine every 12 hours up to and including the morning of day 10. |
| 1 | 49 | Day 1 measurements:   - Video recording of gait ( - Pressure mat measurements |
| 3 | 51 | Day 3 measurements:   - Video recording of gait - Pressure mat measurements |
| 7 | 55 | Day 7 measurements:   - Pressure mat measurements |
| 11 | 59 | General anaesthesia for euthanasaia; necropsy |

^1^ ♂ being an intact male; ^2^ ♀ being an intact female.

2.5.2 Gait measurements

A Footscan® 3D Gait Scientific 2 m pressure mat system (RSscan International, Olen, Belgium) with an active sensor surface of 1.95 m × 0.32 m containing 16384 sensors (2.6 sensors per cm^2^), with a sensitivity of 0.27-127 N/cm^2^ and a measuring frequency of 126 Hz was connected to a laptop with dedicated software (Footscan Scientific Gait 7 gait 2^nd^ generation, RSscan International, Olen, Belgium). Calibration according to the manufacturer’s specifications using a person weighing 62 kg preceded each measuring session. The mat was mounted flush with a 40 cm x 500 cm runway [1]. A holding pen with a swing door was located at both ends of the runway. The entire runway was covered with a rubber mat (5 mm thick, shore value 65° ± 5). All runs were videotaped using 2 action cams (JVC-GC-XA1, JVC, Kawagana-ku, Japan) on both ends of the runway. The cameras were mounted 2 meters above the runway and each faced down to the middle of the runway, while capturing the entire runway ‘in frame’. On measuring days, pigs from one pen were placed in a holding area adjacent to the runway. The pig in the runway was able to hear and smell its pen-mates but could not see them. Pigs were let into the runway one by one and tested in the order they presented themselves in. Two people at either side of the runway operated the guillotine doors and used clickers and chocolate treats to reward the pigs. A third investigator, blinded to treatment, operated the software and judged each run on the following criteria: Pigs had to trot the entire length of the runway at a visually steady pace in either direction in a straight line and looking straight ahead. Runs that fulfilled these criteria were saved and the process was repeated until 3 runs meeting these criteria were collected. On test days, all pigs performed the required 3 valid runs within 5 minutes. Similar to the training sessions, pigs received a click and reward if the run was performed correctly.

2.5.3 Euthanasia

General anaesthesia of the animal was induced under identical conditions as described preceding the intra-articular injections. When clinical depth of anaesthesia was judged as adequate, IV access (ear vein, jugular of superficial abdominal vein) was secured using an IV cannula and the pigs were euthanized by IV injection of 15 ml of Pentobarbitone (400 mg/ml, Euthanimal, Alfasan, Woerden, The Netherlands). After euthanasia, the pigs were transported to the Department of Pathobiology of the Faculty of Veterinary Medicine of Utrecht University.

2.5.4 Necropsy and histopathology

Within hours of euthanasia, a full macroscopic necropsy including opening of left and right carpal joints, shoulder joint, elbow joints, knees and tarsal joints was performed. Samples of ca. 4mm thick were taken of both radiocarpal joints using a K430 band saw (Kolbe, Germany; blades Munkfors, Sweden). These samples were placed in 10% neutral buffered formalin and stored at room temperature until sufficiently fixated. After fixation the samples of the joints were decalcified in 10% ethylene-diamine-tetra-acetic acid (EDTA). Decalcification time varied between 2 and 4 weeks. Hereafter the samples were embedded in paraffin, cut into sections of 3 micrometer, stained with haematoxylin and eosin (HE) and evaluated by light microscopy (Olympus BX-45, Zoeterwoude, The Netherlands) by a board-certified veterinary pathologist.

*2.6. Data preparation*

Claw strikes were manually assigned to left front (LF), right front (RF), left hind (LH) and right hind (RH) limbs using the Footscan software. Stance time (ST; duration of contact of the claw strike (ms) was calculated from the raw data by the program and normalized for body mass. A left-right asymmetry index (ASI) comparing the two front limbs was calculated using the following equations:

| $ASI\left( ST \right)=\frac{\left( ST\left[ LF \right]-ST\left[ RF \right] \right)}{0.5*\left( ST\left[ LF \right]+ST\left[ RF \right] \right)}$ | (S1) |
| --- | --- |

An ASI of 0 indicated perfect symmetry and the extreme values of -200 or +200 indicated non-weight-bearing lameness on the left and right side, respectively.

*2.6. Statistical analysis*

Data were analyzed using IBM SPSS Statistics for Windows, version 21.0. A Kruskal Wallis test was performed to determine if a difference between the test days in the mean ASI(ST), normalized for the baseline was present. A Mann Whitney U test was performed to assess if a difference between MIA treatment and SC treatment was present.

Statistical significance was set at P<0.05. Unless indicated otherwise, results are presented as mean +/- SD.

3. Results

All animals were judged to be clinically healthy and of sound locomotion at the time of recruitment up to the point of the baseline Footscan measurement.

A variable degree of lameness was observed 4-5 hours after intra-articular injection in all animals, upon which they received as per protocol rescue analgesia consisting of 0.01 mg/kg of IM buprenorphine (0.3 mg/ml, Temgesic, Reckitt Bensicker BV, Hoofddorp, Netherlands) every 12 hours. As macroscopic joint morphology was the main parameter of interest in this MIA dose finding study, the rescue analgesia was converted to standard post procedural pain management for all animals for the remainder of the duration of the study (i.e. 10 days), to minimize suffering. Clinical lameness had resolved by day 2 for 2 animals (after unblinding assigned to the SC treatment), while the most clinical lame animals during the 10 days upon unblinding turned out to have been assigned at the 40 mg MIA treatment. One animal developed constipation on day 7 (after unblinding assigned at the 40 mg MIA treatment) which resolved following 2 treatments of a hemicellulose solution via gastric tube on day 7 and the morning of day 8.

3.1. Pathology

3.1.1. Gross Pathology

All 8 animals were found to be in excellent conditions and no visual signs of internal organ changes were observed. In the animal with constipation on day 7, at macroscopy a localized circular redness of the jejunal mucosa was observed. Macroscopically, none of the animals showed changes in opened joints, other than the target joint or their contralateral joint.

In the SC-group the 2 target joints and their respective contralateral carpal joint did not show any macroscopic alterations. In the MIA-10 group, no macroscopic changes to the cartilage in the target joint were observed. However, in one target joint, the dorsal capsule of the carpal joint showed a small hematoma. In the MIA-20 group, the target joint of both animals showed a mild increase in synovial fluid volume, but of apparent normal consistency. The synovial membrane on the dorsal aspect showed increased redness. In one animal, the contralateral carpus showed a subtle thickening of the joint capssule. In the MIA-40 group, in both animals the dorsal aspect of the target joint showed focal red discoloration of cartilage and the adjacent synovial membrane. Both contralateral carpi did not reveal any abnormalities on macroscopy.

3.1.2. Histopathology of target joints

At 11-days following intra-articular injection, no histopathological changes to the cartilage or subchondral bone were observed in the 2 animals of SC-group. In the MIA-10 group, the target joint of both animals showed subacute focal necrosis of the joint cartilage, without histological alterations to the subchondral bone. In the MIA-20 group, a multifocal extensive subacute necrosis of the joint cartilage was found, with an unaltered morphology of subchondral bone at histology. Target joint alterations in the 2 animals of the MIA-40 group comprised of acute large confluent focal necrosis over all captured joint cartilage, sporadically with small areas of exposed subchondral bone. At multiple sites moderate subchondral bone resorption was apparent.

3.2. Gait analyses

Pre-injection, ASI (ST) in all 4 groups were close to 0 (0 indicating perfect symmetry). Compared to the ASI (ST) normalized for baseline in the SC-group, all 3 MIA treatment groups had a decreased ASI (i.e. longer stance time on the contralateral joint). Within each time-point after intra-articular injection, statistical differences between the MIA treatment groups with regards to the magnitude of negative ASI ST were absent. For all three MIA treatment groups, a trend towards normalization of baseline normalized ASI (ST) deviation at day 7 was noted.

4. Discussion and Conclusion

Dose-dependent effects of intra-articular MIA where found on histopathology, while isotonic saline did not induce discernable joint alterations 11 days after intra-articular injection. The histological joint changes observed in the MIA-20 and MIA-40 group 11 days after intra-articular MIA injection are comparable to joint changes reported in rats following a high dose (3 mg) MIA injection [2]. Only the MIA-40 group showed subchondral bone resorption, of already moderate magnitude.

Animals in all MIA groups showed increased asymmetry in stance time, with the left front limb showing a decrease in stance time compared the the right front leg. This is in accordance with Karriker et al [3], which also reported a decrease in ST in the affected limb of lame pigs. Although buprenorphine has been reported to ameliorate spontaneous occurring lameness in pigs [4], in the rescue dose regime used here, buprenorphine did not appear very effective in preventing dynamic weigh bearing changes in MIA-induced osteoarthritis in a single carpal joint. The efficacy of the partial OP3 (mu-) receptor agonist buprenorphine has not been evaluated in MIA-induced osteoarthritis previously. However, in a MIA osteoarthritis model ins rats a single dose of the full OP3 agonist morphine up to 3 mg/kg improved the static, and -to a lesser degree- the dynamic weight bearing [5]. However, this was demonstrated in a more sub-acute phase (3 weeks following MIA-injection), when subchondral bone changes and resultant nociceptive input becomes more dominant [2]. Another study using a single -even higher- dose of morphine (6 mg/kg) demonstrated good efficacy on locomotion when administered on day 3 as well as on day 20 following intra-articular MIA in rats. However, these high dosages are not advisable as daily rescue analgesic dose. Despite decreased gut motility could have originated from uncontrolled pain, the temporary obstipation seen in one of the animals does caution for higher dose-regimes for buprenorphine when administered daily for more than a week. The rescue analgesic dose regime of buprenorphine in this pilot study was based on a buprenorphine dose used for post-operative pain relief [6]. Although the dose-regime was likely not adequate to effectively improve dynamic weight bearing after intra-articular MIA, it may have been partially effective in improving static weight bearing.

The results of this pilot study indicate that intra-articular MIA induces lameness in pigs for at least 7 days as demonstrated by objective kinetic data on stance time during locomotion. The dose of intra-articular MIA of 20 mg for the main experiment was based on the histopathology data obtained in the pilot study. For the main study, animals would have to remain in the study protocol for at least 56 days following MIA-injection. With the 40 mg dose, moderate subchondral bone resorption was already observed at day 11, thus increasing the change that a humane endpoint would be reached for some animals before day 56. We aimed for a dose of MIA in our main experiment to result in a sustained, progressively developing osteoarthritis. Because we observed more severe osteoarthritic joint changes including involvement of the synovial membrane with a dose of 20 mg, we judged this dose most suitable for our main experiment.

**Author Contributions:** Conceptualization, J.U., E.M. and F.J.S; Methodology, E.M., J.U. and FJS; Formal analysis, E.M. and F.J.S.; Investigation, J.U. and E.M; Resources F.J.S.; Writing—original draft preparation, J.U., E.M. and F.J.S.; writing—review and editing, E.M., J.U. and FJS.; visualization, E.M., F.J.S and J.U.; Supervision, F.J.S.; Project administration, F.J.S.;

**Funding:** Please add: “This research received no external funding”

**Acknowledgments:** The authors wish to express their gratitude and acknowledge the substantial contributions towards animal care, training the animals and assistance in conducting the experimental measurements: Ms E.C.A Waas, Mr. M Kranenburg, A. van Dam and Ms M.M.M. Wetzels.

**Conflicts of Interest:** “The authors declare no conflict of interest.”

References

1. Meijer, E.; Oosterlinck, M.; Nes, A. van; Back, W.; Staay, F.J. van der Pressure mat analysis of naturally occurring lameness in young pigs after weaning. *BMC Vet. Res.* **2014**, *10*, 193.

2. Guingamp, C.; Gegout-Pottie, P.; Philippe, L.; Terlain, B.; Netter, P.; Gillet, P. Mono-iodoacetate-induced experimental osteoarthritis: a dose-response study of loss of mobility, morphology, and biochemistry. *Arthritis Rheum.* **1997**, *40*, 1670–1679.

3. Karriker, L.A.; Abell, C.E.; Pairis-Garcia, M.D.; Holt, W.A.; Sun, G.; Coetzee, J.F.; Johnson, A.K.; Hoff, S.J.; Stalder, K.J. Validation of a lameness model in sows using physiological and mechanical measurements. *J. Anim. Sci.* **2013**, *91*, 130–136.

4. Meijer, E.; van Nes, A.; Back, W.; van der Staay, F.J. Clinical effects of buprenorphine on open field behaviour and gait symmetry in healthy and lame weaned piglets. *Vet. J. Lond. Engl. 1997* **2015**, *206*, 298–303.

5. Ishikawa, G.; Nagakura, Y.; Takeshita, N.; Shimizu, Y. Efficacy of drugs with different mechanisms of action in relieving spontaneous pain at rest and during movement in a rat model of osteoarthritis. *Eur. J. Pharmacol.* **2014**, *738*, 111–117.

6. Tranquilli, W.J.; Thurmon, J.C.; Grimm, K.A. Chapter 29- Swine. In *Veterinary Anesthesia and Analgesia (Fourth Edition)*; Blackwell Puyblishing: Oxford, 2007; pp. 747–763 ISBN 978-0-7817-5471-2.

| 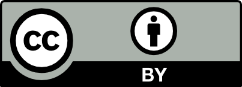 | © 2019 by the authors. Submitted for possible open access publication under the terms and conditions of the Creative Commons Attribution (CC BY) license (http://creativecommons.org/licenses/by/4.0/). |
| --- | --- |

Material C: Representative macroscopic intercarpal joint morphology 68 days following an intra-articular injection of isotonic saline or 20 mg Monosodium Iodoacetate.

| 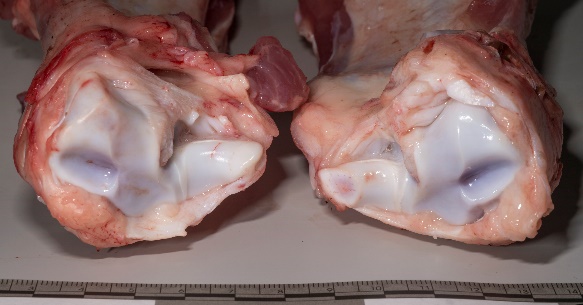  (**a**) | 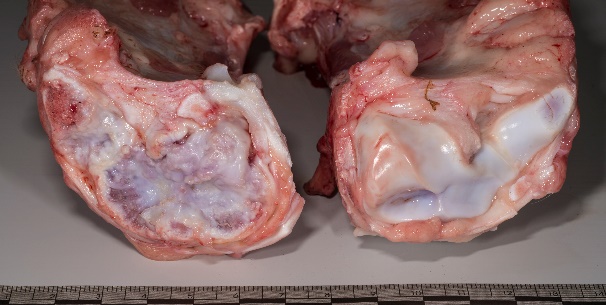  (**b**) |
| --- | --- |

**Figure S4.** Macroscopic presentation of the proximal aspect (looking in a ventro-dorsal direction) of the intercarpal joint on necropsy. (**a**) Intercarpal joint of an animal from the Saline Control group, with the target joint on the left and the contralateral joint on the right. Note that the cartilage is white, smooth and shiny on presentation; (**b**) Intercarpal joint of an animal from the MIA-group, with the target joint on the left and the contralateral joint on the right. Note the striking difference with the contralateral joint, with the target joint showing an even plane due to cartilage being largely absent, subcortical bone collaps and displacement with red fibrous connective tissue.
