# Supplementary figures and images for: A Monosodium Iodoacetate Osteoarthritis Lameness Model in Growing Pigs
Source: Animals (Basel). 2019 Jul 1;9(7):405. doi: 10.3390/ani9070405 (PMC6680622; doi:10.3390/ani9070405)

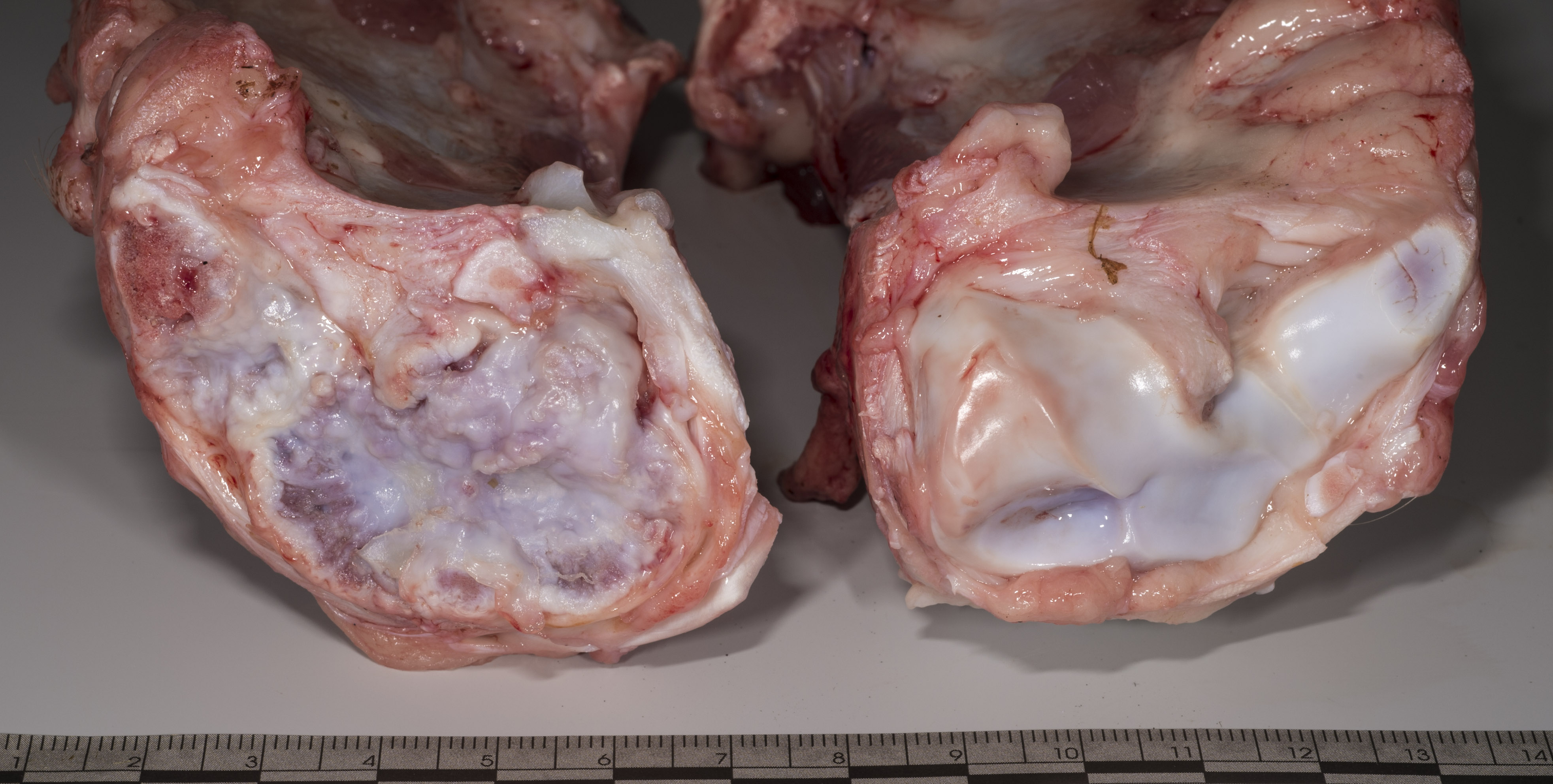

Supplement: Supplementary file 1 [file animals-09-00405-s001.zip › Uilenreef 30-6-2015 06-55.jpg]

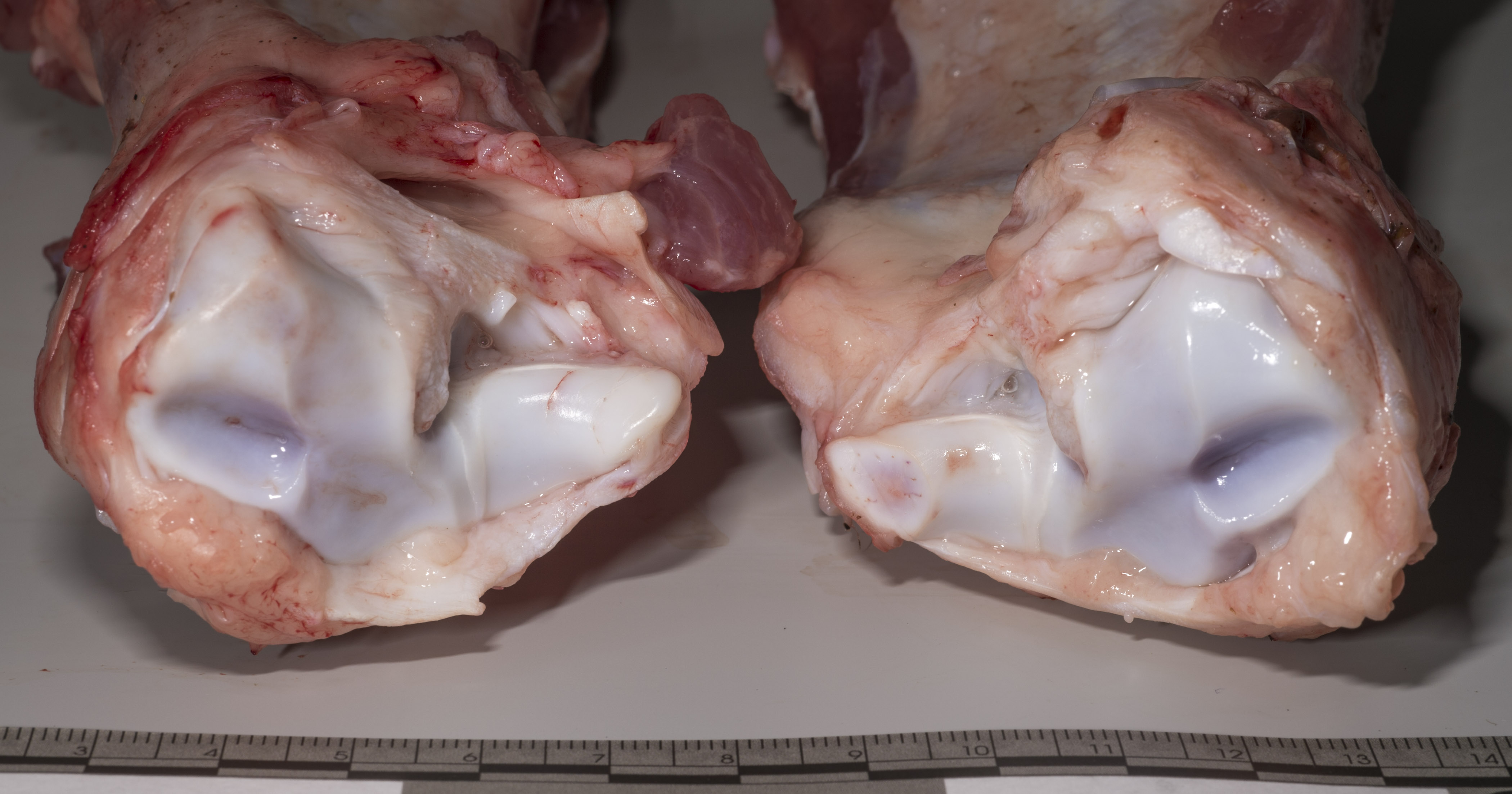

Supplement: Supplementary file 1 [file animals-09-00405-s001.zip › Uilenreef 30-6-2015 10-54.jpg]
